# Comparison of Melioidosis Indirect Hemagglutination Assay between Three Testing Laboratories in Australia

**DOI:** 10.1128/spectrum.04949-22

**Published:** 2023-03-27

**Authors:** Mark Armstrong, Justin Morgan, Olena Kazey, Kevin Freeman, Robert Norton

**Affiliations:** a Pathology Queensland, Townsville University Hospital, Townsville, Queensland, Australia; b PathWest Laboratory Medicine, Nedlands, Western Australia, Australia; c Territory Pathology, Royal Darwin Hospital, Darwin, Northern Territory, Australia; d Faculty of Medicine, University of Queensland, Brisbane, Queensland, Australia; Taichung Veterans General Hospital

**Keywords:** melioidosis, Australia, IHA, serology

## Abstract

Melioidosis caused by Burkholderia pseudomallei causes significant morbidity and mortality in Southeast Asia and northern Australia. Clinical manifestations remain diverse, including localized skin infection, pneumonia, and chronic abscess formation. Culture remains the gold standard of diagnosis, with serology and antigen detection tests playing a role if culture is unfeasible. Serologic diagnosis remains challenging, with limited standardization across different assays. In areas of endemicity, high rates of seropositivity have been documented. The indirect hemagglutination assay (IHA) is one of the more widely used serologic tests in these areas. In Australia, only three centers perform the test. Annually, laboratory A, laboratory B, and laboratory C perform approximately 1,000, 4,500, and 500 tests, respectively. A comparison of a total of 132 sera was analyzed from the routine quality exchange program between these centers from 2010 until 2019. Overall, 18.9% of sera tested had an interpretative discrepancy between laboratories.

**IMPORTANCE** This study found significant discrepant results between three Australian centers offering the melioidosis indirect hemagglutination assay (IHA), despite testing the same samples. We have highlighted that the IHA is a nonstandardized test, which had different source antigens at each of the different laboratories. Melioidosis is a global disease, is associated with significant mortality, and is perhaps under recognized. It is likely to have increasing impact with changing weather patterns. The IHA has been used frequently as an adjunct to the diagnosis of clinical disease and is the mainstay of determining seroprevalence within populations. Despite its relative ease of use, especially in low resource settings, our study highlights the significant limitations of the melioidosis IHA. It has wide ranging implications, serving as an impetus for developing better diagnostic tests. This study is of interest to practitioners and researchers working in the various geographic regions affected by melioidosis.

## INTRODUCTION

Melioidosis is caused by the environmental bacterium Burkholderia pseudomallei. The disease causes significant morbidity and mortality, especially in regions of high endemicity, such as Southeast Asia and northern Australia (1). Clinical presentations are highly variable and include focal skin infections, pneumonia, and chronic abscesses along with a wide range of other manifestations (2, 3). The diagnosis of melioidosis is challenging, with culture being the gold standard. However, the use of this method may not be feasible, especially where blood cultures remain negative, such as in the setting of chronic disease or where a deep-seated abscess may prove difficult to get a sample from. Therefore, serology and antigen detection tests still play an important adjunct role in diagnosis. Specific difficulties remain with serologic testing in melioidosis, including limited standardization across different assays (4). In addition, high rates of background seropositivity are observed in areas of endemicity in otherwise healthy people (5).

The indirect hemagglutination assay (IHA) is one of the more widely used serologic tests in areas of endemicity (6). It is relatively easy to perform in resource-limited settings. Despite this benefit, different laboratories show significant variation in the strains and protocols they use for antigen preparation, which makes it difficult to compare testing between these different centers. Furthermore, to add to this issue, the IHA is a laboratory-developed test and there are currently no commercial melioidosis serologic assays available. In Australia, there are three laboratories that perform melioidosis IHA. These laboratories collectively test all requests within Australia, receiving isolates from different regions of endemicity and, on occasion, from overseas. These laboratories are described as laboratory A, laboratory B, and laboratory C, and each laboratory performs approximately 1,000, 4,500, and 500 melioidosis IHA tests annually, respectively (personal communication with R. Norton, K. Freeman, and J. Morgan).

As part of a quality assurance exchange program (QAP), serum samples are distributed between these three laboratories. Sera from patients known to have culture confirmed melioidosis were selected by the supervising serologic scientist at the sending lab to be tested by all the laboratories. This QAP allows the comparison of both the qualitative and titer results of testing performed on the same sera, in the different laboratories. Three dispatches are sent out (one from each participating laboratory) each year, with typically 4 to 6 sera per dispatch. We describe the results of this QAP over the 10-year period from 2010 until 2019.

## RESULTS

Over the period from 2010 to 2019, a total of 26 separate dispatches of sera were reviewed. Each dispatch comprised between 4 and 6 samples of sera from separate patients (excluding 2 duplicate sera). A total of 132 sera were tested during this period across all three laboratories. Laboratory C did not participate in dispatch 2 and 3 of 2018, so a further 11 samples were tested by laboratory A and laboratory B (total of 143). Transport delays from the sending laboratory were noted for dispatch 3 in 2010, with potential relative increased titer results for the sending laboratory. With this same dispatch, there was also concern about one sample having anti-sheep antibody present. Table 1 outlines dispatch details.

**TABLE 1:** Sample dispatches

Yr	No. of dispatches sent	Total no. of sera tested at each laboratory		
		Laboratory A	Laboratory B	Laboratory C
2010	3	17	17	17
2011	3	16	16	16
2012	3	17	17	17
2013	2	11	11	11
2014	2	11	11	11
2015	3	18	18	18
2016	3	15	15	15
2017	2	11	11	11
2018	3	17	17	6
2019	2^*a*^	10	10	10
Totals	26	143	143	132

a Year not completed at the time of review.

Samples tested at all three laboratories (*n* = 132) were assessed for the degree of discrepancy between laboratories. Of these samples, 64 (48.5%) had at least a 4-fold difference in titers between centers (excluding lower limit titers, <). Of all sera tested, a total of 25 had discrepant interpretations between laboratories (e.g., 1 reported a negative result based on a titer, while another reported a positive or borderline result). Of all sera tested, the degree of discrepancy was 18.9% (25/132), as there were no noted discrepancies for dispatch 2 and 3 (during 2018), which were tested only at laboratory A and B (Table 2). These differences between laboratories are shown in Table 3, with titer results and interpretative criteria applied by the testing laboratory. To help clarify these differences, a result was considered an “outlier” if it disagreed with the other two laboratories. If all three laboratories had disagreement, all three results were considered outliers. To further determine the degree of disparities, absolute titer results where a defined endpoint was reached were compared. This comparison was done as it is likely that titer differences greater than a 2-fold difference represent systematic differences rather than random error. Discrepant results with a titer difference of 4 or more were tallied, with 36.0% (9/25) reaching this degree of difference. These results are outlined in Table 4. A total of 33 of the 143 (23.0%) sera tested were nonreactive in all the laboratories that testing them. Furthermore, while not representing continuous data, the median titer of samples tested at laboratory A was 80 (interquartile range, [IQR], 5 to 640), that for laboratory B was 160 (IQR, 20 to 800), and that for laboratory C was 320 (IQR, 320 to 2,560). Laboratory A had 79 reactive sera (sensitivity, 59.8%), laboratory B had 94 reactive sera (sensitivity, 71.2%), and laboratory C had 93 reactive sera (sensitivity, 70.5%).

**TABLE 2:** Sample results

Yr	No. of discrepant results	No. (%) of total sera tested
2010	5	5/17 (29.4)
2011	3	3/16 (18.8)
2012	4	4/17 (23.5)
2013	2	2/11 (18.2)
2014	0	0/11 (0)
2015	6	6/18 (33.3)
2016	3	3/15 (20.0)
2017	0	0/11 (0)
2018	1	1/6 (16.7)^*a*^
2019	1	1/10 (10.0)
All yrs:	25	25/132 (17.5)

a Total sera listed as total tested between all three laboratories. Laboratory C did not test dispatches 2 and 3 (11 sera), but no discrepant results were found between these 11 sera tested only between laboratory A and laboratory B.

**TABLE 3:** Discrepant results

Result no.	Titer, interpretation by laboratory^*a*^		
	A	B	C
1	<5, nonreactive	**40, reactive**	<20, nonreactive
2	**<5, nonreactive**	40, reactive	80, reactive
3	**<5, nonreactive**	80, reactive	640, reactive
4	**<5, nonreactive**	80, reactive	320, reactive
5	**<5, nonreactive**	**20, borderline**	**80, reactive**
6	20, borderline	20, borderline	**640, reactive**
7	10, nonreactive	<20, nonreactive	**80, reactive**
8	<5, nonreactive	<20, nonreactive	**80, reactive**
9	160, reactive	320, reactive	**<20, nonreactive**
10	<5, nonreactive	**320, reactive**	10, nonreactive
11	**10, nonreactive**	40, reactive	640, reactive
12	**10, nonreactive**	320, reactive	2560, reactive
13	**20, borderline**	320, reactive	40, reactive
14	20, borderline	20, borderline	**40, reactive**
15	10, nonreactive	**80, reactive**	20, nonreactive
16	10, nonreactive	**40, reactive**	20, nonreactive
17	**20, borderline**	80, reactive	80, reactive
18	<5, nonreactive	**20, borderline**	<20, nonreactive
19	**<5, nonreactive**	160, reactive	320, reactive
20	**10, nonreactive**	80, reactive	80, reactive
21	<5, nonreactive	**20, borderline**	<20, nonreactive
22	40, reactive	640, reactive	**<20, nonreactive**
23	**20, borderline**	40, reactive	80, reactive
24	<5, nonreactive	**20, borderline**	20, nonreactive
25	**20, borderline**	5,120, reactive	40, reactive

a Outlier (as defined in the text) results are highlighted by bold text at lab B titers down to 1:10 but are routinely listed as <20 in QAP worksheets and at lab C titers down to 1:5 but are mostly listed as <20 in QAP worksheets.

**TABLE 4:** Discrepant results outlier interpretation

Result	No. of results by laboratory		
	A	B	C
Reactive	0	4	4
Nonreactive	7	0	2
Borderline	4	3	0
Complete disagreement	1		
4-Fold difference or greater^*a*^	9		

a In reactive defined titers, with < titers excluded.

## DISCUSSION

First described over 50 years ago, the IHA remains in widespread use (7). Ease of use, familiarity, and relative low cost are likely reasons for its widespread use, despite the development of increasingly better and less time-consuming diagnostic methods (8). Patients with culture-confirmed melioidosis have been shown to not seroconvert their IHA results in acute illness, suggesting a limited diagnostic utility in this setting (9). Interpretation of the test is further complicated in areas of endemicity, due to high levels of background seroconversion in otherwise healthy populations, with Chaichana et al. (6) showing IHA seropositivity of 38% (≥1:80) in an endemic area in Thailand (10). In addition to this finding, they demonstrated poor IHA sensitivity in patients with B. pseudomallei bacteremia. In all cases of culture-confirmed melioidosis, there was significant variation of seroconversion patterns, with 3% of patients having a transient seroconversion (6). Given these significant limitations, the greatest utility of the IHA may be in travelers who normally reside in countries with no endemicity returning from an area with endemicity. This would help support the diagnosis of melioidosis, although a definitive diagnosis would still require a positive culture (11).

While clinical data were not available for the patients in this study, it is likely that the melioidosis IHA will continue to be requested by clinicians for a range of clinical presentations and across a broad geographic area. Notwithstanding the concerns mentioned, the lack of standardization between centers performing the test further decreases the diagnostic utility and epidemiologic interpretation of serially obtained IHAs. Further testing of the same serum sample may result in discrepant titer levels if tested in different laboratories.

As far as we are aware, this report is the first study of a comparison of three different laboratories testing the same samples from patients with known melioidosis. Our findings highlight the lack of reproducibility with 64 of the 132 (48.5%) samples tested at all 3 labs having a 4-fold difference or more between laboratories. This result led to 25 discrepant results (18.9% of the total) by using the defined interpretative cutoff criteria. It is likely that some systematic differences existed with the IHA testing between labs, as shown by the median titers between the different laboratories ranging from 80 to 320. These differences most likely occur because of the different melioidosis isolates used in the preparation of crude antigen. Another possible source of variation between laboratories could be the different strains of Burkholderia pseudomallei isolated from different geographic regions. Overall, there were very limited technical issues noted for testing apart from one dispatch having transport delays and concern over anti-sheep antibodies. It is possible that these issues could have altered results, but they should not have resulted in the systematic differences that were observed.

This study has limitations. First, it is a small study between only three different laboratories in a single country. Isolates came only from a limited geographic area, although it was not defined, as this information was unavailable. It is possible that melioidosis isolates from distinct geographic regions will have a significant impact on IHA discrepancies. Second, these results are retrospective, and techniques were not standardized across laboratories with no overall oversight of them. Serologic testing was performed by multiple operators, and it is possible that unrecorded errors or differences in interpretation occurred as a result. Despite these limitations, all laboratories had well-defined standard operating procedures, and testing was conducted by experienced scientists well used to performing and interpreting the test. Third, there were no clinical or microbiological details of patients from whom the sera were obtained. While all patients were culture positive, information such as site of infection and clinical severity was not available. Further analysis and a determination of the significance of differences between sites, therefore, were not able to be performed. Harris et al. (12) demonstrated diabetes as a positive predictive factor in having positive IHA results in patients with culture-confirmed melioidosis. Unknown clinical and demographic differences between sites could have confounded our results.

A multitude of new serologic tests are being developed for the serodiagnosis of melioidosis, utilizing different antigens and with variable reported performance parameters (13–15). Up to 26% of patients have been shown to not seroconvert with IHA, which is a concerning finding given its widespread use (12). In this review, 33 of the 143 (23.0%) sera tested did not react in any of the laboratories that tested it, which is in keeping with some patients not seroconverting despite having confirmed melioidosis, although the time frame of the IHA in relation to their illness is not defined. The sensitivities of the assays in this study were relatively low, ranging from 59.8% to 71.2%, which is in keeping with the relatively large percentage of nonreactive samples. Some recombinant-based assays in an immunochromatographic and enzyme-linked immunosorbent assay (ELISA) formats show good sensitivity in the range of 88.3 to 93.7% and specificity of 86.1 to 100% (8, 16). These results compare favorably to the IHA (using a titer of ≥1:160) performed on patients and healthy donors in Thailand, having a sensitivity of 69.5% in patients and specificity in healthy donors of 67.6% (16).

While the role of these newer tests is yet to be defined, it is likely that they will not have the same issues with standardization that are experienced with testing modalities (such as the IHA) using crude antigen extraction. Even with improved standardization of the IHA across different laboratories, it is likely that these newer assays will have a greater ability to identify acute infection than IHA (17). Specifically, assays using the Hcp1 antigen have high titers early in disease, with an observed decline in levels over time, suggesting possible utility in monitoring disease progress (17).

In conclusion, this study highlights significant disparity between laboratories in a single country undertaking IHA testing on shared sera for patients with confirmed melioidosis. It calls into question the utility of the IHA as a diagnostic test for melioidosis, especially when newer recombinant immunochromatographic and ELISAs are becoming increasingly available.

## MATERIALS AND METHODS

Records for all available sera tested as part of the QAP were included in the study. Data were abstracted into an excel spreadsheet and included the date of testing, titer result, interpretation of result, and noted discrepancies. Any difficulties with the testing process were also described, such as delays in transit or anti-red blood cell antibodies. Testing was performed during routine workflow by medical laboratory staff with results recorded onto paper-based worksheets and then had subsequent cross-checking by another staff member before data were entered into the laboratory electronic information system. Sera to be tested from melioidosis patients had no recorded clinical details.

### Test principle.

The IHA uses washed sheep red blood cells, sensitized by incubation with melioidosis antigen. A second step washes away any unattached antigen. Inactivation of serum is achieved by heating it to a defined temperature in saline. Incubation with nonsensitized sheep red cells removes nonspecific agglutinins. Sample sera are diluted by 2-fold serial dilutions on a 96-microtiter plate. The antigen-sensitized red cells are then added to the wells and allowed to incubate. Melioidosis antibodies cause hemagglutination and formation of a “lattice” if a reaction occurs. The diluted serum allows the semiquantification of the melioidosis antibody.

### Preparation of melioidosis antigen.

Laboratory A used 5 isolates in preparation of antigen material for the IHA. Isolate 1 was a National Collection of Type Culture (NCTC) isolate number 13178. This isolate was collected from an Australian resident with neuromelioidosis from the same state of the laboratory. Isolate 2 was a NCTC isolate number 13179 from a skin swab of a resident from the same state. Isolate 3 was from a blood culture of a resident of the same state. Isolate 4 was from a sputum sample of a resident of the same state. Isolate 5 was from a urine isolate of an Australian resident in a different state.

Laboratory B uses 3 isolates in the preparation of antigen material for the IHA. All 3 isolates were from clinical samples obtained from Australian residents within the same state as the laboratory. Clinical details were not available. The same antigen strains were used throughout the duration of the study period.

Laboratory C uses 3 isolates in the preparation of antigen material for the IHA. Isolate 1 was an NCTC isolate, number 10276, from a patient infected in India. Isolate 2 was an NCTC isolate, number 13177, isolated from an Australian resident of the same state as the laboratory. Isolate 3 was collected from a blood culture in an Australian resident from a different state.

Preparing the melioidosis antigen and sourcing sheep red cells were done individually at each laboratory, and therefore, significant variation of the antigen extract can occur due to different geographically sourced strains. Also, the information available for the isolates of melioidosis sourced by each laboratory was available for the current protocols. It is possible that different source isolates were used at each of laboratories over the time of the audit (2010 to 2019), but unfortunately, this information is not available for each of the laboratories. All testing is run with a negative, low-positive and a high-positive control. Melioidosis antigen preparation was performed prior to individual runs of IHA, using in-house methods of crude antigen extraction. All laboratories prepared antigen in bulk to last several years, with a defined shelf life of 3 years. Minor differences in technique for antigen extraction was noted for each laboratory; however, all laboratories checked isolates for purity by subculture onto horse blood and MacConkey agar plates. All isolates had identification confirmed by phenotypic methods. All laboratories incubated the antigen preparation strains for 2 weeks at 35°C (or 37°C for laboratory C), with mixing and using protein-free liquid media. The same inactivation technique (autoclaving a flask of antigen preparation material at 121°C for 15 min) was used in each laboratory. The antigen concentration was achieved by centrifugation, which was defined as 20,000 rpm for 30 min for both laboratory B and C. Phenol at 0.5% by volume was added to the antigen supernatant for each lab. All laboratories performed titration of the prepared antigen to determine the optimum dilution of each new batch by performing block titration against a positive serum of known titer. Aliquots of antigens are stored at −70°C until they are ready for use. Detailed descriptions of this test are available within performing laboratory standard operating procedures (internal documents). These methods are based on work published previously (7, 18–21).

### Test procedure.

As noted, the methods for each laboratory were essentially identical except for the dilution number performed. Laboratory A performs serial doubling dilutions for the IHA of 1:5 up to 1:5,120, after which results are designated greater than (>). Laboratory B performs serial doubling dilutions starting at 1:10, with no defined upper limit. Laboratory C has serial doubling dilutions of 1:5 up to 1:10,240, after which results are designated greater than (>). Interpretation criteria differed slightly, which are detailed as follows: laboratory A, <1:20 nonreactive, 1:20 borderline, >1:20 reactive; laboratory B, <1:20 nonreactive, 1:20 borderline, >1:20 reactive; and laboratory C: <1:40 nonreactive, ≥1:40 reactive.

These cutoff values are similar to that suggested in the literature with ≥1:40 often being used to define positive results in the Australian setting (22). The formation of a “lattice,” rather than a “button” at the bottom of the well, forms the reading point from which the titer is determined visually. While there is some subjectivity in the interpretation of this endpoint, each laboratory had internal training (including visual examples) to minimize the inconsistency of plate reading and recording of the endpoint. The interpretive criteria did not change over the study period for any of the laboratories.

### Ethics.

Laboratory data custodian approval was approved, with reference number CT_5057. All data were retrospective and deidentified, and patients were not contacted at any time during the study.

## Supplementary Material

Reviewer comments
